# Effect of using pump on postoperative pleural effusion in the patients that underwent CABG

**DOI:** 10.5935/1678-9741.20150029

**Published:** 2015

**Authors:** Mehmet Özülkü, Fatih Aygün

**Affiliations:** 1 Başkent University, Konya Research and Medical Center, Turkey.

**Keywords:** Coronary Artery Disease, Cardiac Surgical Procedures, Cardiopulmonary Bypass, Pleural Effusion, Coronary Artery Bypass, Off-Pump

## Abstract

**Objective:**

The present study investigated effect of using pump on postoperative pleural effusion in patients who underwent coronary artery bypass grafting.

**Methods:**

A total of 256 patients who underwent isolated coronary artery bypass grafting surgery in the Cardiovascular Surgery clinic were enrolled in the study. Jostra-Cobe (Model 043213 105, VLC 865, Sweden) heart-lung machine was used in on-pump coronary artery bypass grafting. Off-pump coronary artery bypass grafting was performed using Octopus and Starfish. Proximal anastomoses to the aorta in both on-pump and off-pump techniques were performed by side clamps. The patients were discharged from the hospital between postoperative day 6 and day 11.

**Results:**

The incidence of postoperative right pleural effusion and bilateral pleural effusion was found to be higher as a count in Group 1 (on-pump) as compared to Group 2 (off-pump). But the difference was not statistically significant [*P*>0.05 for right pleural effusion (*P*=0.893), *P*>0.05 for bilateral pleural effusion (*P*=0.780)]. Left pleural effusion was encountered to be lower in Group 2 (off-pump). The difference was found to be statistically significant (*P*<0.05, *P*=0.006).

**Conclusion:**

Under the light of these results, it can be said that left pleural effusion is less prevalent in the patients that underwent off-pump coronary artery bypass grafting when compared to the patients that underwent on-pump coronary artery bypass grafting.

## Abstract (pt)

**Introdução:**

O presente estudo investigou efeito da utilização de bomba em derrame pleural pós-operatório nos casos de pacientes que se submeteram à cirurgia de revascularização miocárdica.

**Métodos:**

Um total de 256 pacientes que foram submetidos à cirurgia de revascularização isolada no ambulatório de Cirurgia Cardiovascular foram incluídos no estudo. Máquina coração-pulmão Jostra-Cobe (Modelo 043213 105, VLC 865, Suécia) foi utilizada em cirurgia de revascularização miocárdica com circulação extracorpórea. Cirurgia de revascularização miocárdica sem circulação extracorpórea foi realizada utilizando Octopus e Starfish. Anastomose proximal na aorta, em ambas as técnicas, foi realizada por grampos laterais. Os pacientes receberam alta do hospital entre os dias 6 e 11 de pós-operatório.

**Resultados:**

A incidência de derrame pleural à direita pós-operatória e derrame pleural bilateral encontrada foi mais elevada em contagem do Grupo 1 (com circulação extracorpórea) em relação ao Grupo 2 (sem circulação extracorpórea). Mas a diferença não foi estatisticamente significativa [*P*>0,05 para derrame pleural à direita (*P*=0,893), *P*>0,05 para derrame pleural bilateral (*P*=0,780)]. O derrame pleural esquerdo encontrado foi menor no Grupo 2 (CEC). A diferença foi estatisticamente significativa (*P*<0,05, *P*=0,006).

**Conclusão:**

Sob a luz destes resultados, pode-se dizer que derrame pleural esquerdo é menos prevalente nos pacientes que foram submetidos à revascularização do miocárdio sem circulação extracorpórea em comparação com os pacientes que foram submetidos à revascularização do miocárdio com circulação extracorpórea.

**Table:** 

**Abbreviations, acronyms & symbols**	
CABG	Coronary artery bypass grafting
COPD	Chronic obstructive pulmonary disease
CAD	Coronary artery disease
BMI	Body mass index
ml	Milliliter
cm	Centimeter
mm	Millimeter

## INTRODUCTION

Coronary artery bypass surgery (CABG) is one of the operations most frequently performed all over the world. Conventional CABG is performed by using cardiopulmonary bypass (CPB) device and called as on-pump CABG, whereas CABG performed without using CPB is called as off-pump CABG. Although postoperative complications are never desirable, they may sometimes be inevitable for some patients. Pleural effusion following CABG is still being encountered despite all efforts of cardiovascular surgeons.

It has been reported that pleural effusion occurs in 41%-87% of the patients in the postoperative very early period^[1-4]^. Pleural effusions that occur following coronary bypass surgery can be classified according to the development period as very early period (within postoperative one week) (perioperative), early period (between postoperative one week and one month), late period (between postoperative two and 12 months), and very late period (after postoperative 6 months, permanent)^[5]^.

It is thought that many factors ranging from surgical technique to the preoperative medications have a role on the development of pleural effusion in postoperative early period. Primarily, there are two basic reasons for pleural effusions that occur in the period so-called perioperative period, which comprises postoperative first one-week. The first is diaphragm disorder and the second is harvesting LIMA^[6-10]^. Heart failure after CABG decreased cardiac output after surgery; pleural infection, pulmonary embolus, and chylothorax are among the causes of postoperative pleural effusion^[5,11]^.

The present study investigated effect of using pump on postoperative pleural effusion in the cases that underwent CABG, as well as the statistical significance of this effect.

## METHODS

### Clinical Characteristics of Patients

A total of 256 patients, who underwent isolated CABG surgery in the Cardiovascular Surgery clinic and had no valvular pathology or connective tissue disease (Marfan syndrome, etc.) were enrolled in the study. The data were retrospectively collected.

In the preoperative period, all patients were questioned in terms of medical history and underwent detailed physical examination. Standard preoperative laboratory tests were performed in the preoperative period in CVS clinic; pulmonary function test (Spirobank Spirometry, MIR medical International Research Product) was performed in case any pathology was detected in the patients during respiratory system anamnesis or examination, transthoracic echocardiography (TTE) (Acuson, Mountain View, Acuson Sequoia C256) was performed in all patients, and bilateral carotid artery Doppler ultrasonography (Toshiba XARIO prime ultrasound) was performed in the patients with pathology detected on carotid artery and peripheral artery examination as well as in the patients that had lesion in the main coronary artery.

Patients were considered as low-weight if body mass index (BMI) was lower than 20 kg/m^2^, normal-weight if BMI was between 20 kg/m^2^ and 24.9 kg/m^2^, over-weight if BMI was between 25 kg/m^2^ and 29.9 kg/m^2^, and obese if BMI was equal to or higher than 30 kg/m^2^.

In the preoperative period, clopidogrel (if receiving) therapy was discontinued five days and acetylsalicylic acid therapy was discontinued three days before surgery in the all patients that would undergo On-Pump (with CPB) CABG and Off-Pump (Beating-heart) CABG.

### Study Groups

The patients that underwent CABG were dichotomized according to two different surgical techniques. The first group (Group 1) consists of patients who underwent CABG by CPB (On-Pump) device. The second group (Group 2) consists of patients who underwent CABG by beating heart (Off-Pump) technique. Proximal anastomoses were performed using side clamps in all patients. Duration of cross-clamping did not exceed 90 minutes and duration of bypass did not exceed 120 minutes in the patients who underwent CABG by CPB cross-clamp technique. All patients underwent surgery by the same surgical team. In order to create a homogeneous group, dialysis patients or the patients with creatinine level over 2 gr/dl, patients with aortic pathology detected during surgery and thereby surgery procedure was changed, patients who had undergone surgery as emergency cases, patients who underwent redo–CABG, patients with postoperative lower respiratory tract infection, patients who developed postoperative diaphragm paralysis, patients who had been re-explored because of drainage, and the patients who died were not included in the study.

### Surgical method

All patients underwent isolated CABG surgery by cardiopulmonary bypass (CPB) device, beating heart technique or beating heart technique under CPB support. Induction of anesthesia was performed with fentanyl, midazolam and pancuronium bromide. Standard median sternotomy was performed and LIMA and other vascular conduits were prepared before CPB has started. After administering 300 IU/kg heparin, CPB was started by roller pump using standard aortic and two-stage venous cannula. All patients initially received high-potassium crystalloid and then cold standard crystalloid cardioplegia and, at the end, hot-shot cardioplegia during surgery. Whilst the left internal mammary artery (LIMA) was used in all of the cases, the right internal mammary artery was not used. Meticulous aseptic technique was performed in the surgery. Unnecessary use of electro-cautery and unnecessary perfusion in CPB (Luxury) were avoided. Heparinization was performed using 150 IU/kg heparin in the patients who underwent surgery with beating heart technique. Distal anastomoses were performed using Octopus and Starfish. Anastomoses to the aorta were performed using side clamps both in on-pump and off-pump technique.

In on-pump or off-pump CABG patients; the left pleura was standardly opened either while preparing LIMA during surgery or just after the LIMA has been prepared. Cold isotonic solution (slash) was used for local cold effect in all on-pump CABG patients. The right pleura was opened in all patients who underwent off-pump CABG. At the end of on-off pump CABG procedure, mediastinal and pleural drains were inserted through the subxiphoid area. Inserting mediastinal and left and right pleural drains through the subxiphoid area is a standard procedure in our clinic.

### Postoperative Care

Under normal conditions in the postoperative period, acetylsalicylic acid (Coraspin 300^®^) was commenced at a dose of 300 mg/day together with enteral nutrition in all patients in order to reduce the risk of complication after CABG. Cefazolin sodium (Cefamezin^®^-IM/IV), which is used as standard prophylactic antibiotic in our clinic, was administered at a dose of 1gr for once 30 minutes before surgery and then continued for 72 hours after surgery at a dose of 1g at 8 hours intervals. Postoperative blood glucose regulation in diabetic patients was strictly done using insulin glargine 100 IU/ml (Lantus^®^ flacon) and human soluble regular insulin 100 IU/ml (Humulin-R^®^ flacon). Insulin infusion was not avoided when needed. Blood glucose concentration was kept at the level of 200 mg/dl in all patients.

In the postoperative period, the patients stayed at CVS intensive care unit for 48 hours and then they were admitted to the CVS clinic after removing the drains (thoracic and mediastinal drains; they were kept until the drainage became serous and amount of drainage in the last 5 hours was 50 cc) and arterial catheters in the third 24 hours. Central vascular line was removed on the 4^th^ postoperative day in the CVS clinic. The patients were discharged on the 6^th^-11^th^ postoperative days and checked for pleural effusion on the 7^th^ postoperative day.

After they were admitted to the CVS clinic, pleural fluid was controlled with a posteroanterior chest radiography until discharged by 24 hours interval. Patients were evaluated with ultrasonographic costophrenic angle blunting. The patients who presented excessive pleural liquid (500 cc and above) were fitted Pleurocan (8-10French-B. Braun, Melsungen, Germany) If the mount of the fluid taken from the pleural space was 500 cc or above, it was considered as pleural effusion.

### Statistical Analysis 

Statistical analyses were performed by SPSS program (SPSS Inc., Chicago, IL, USA). Statistical significance of nonparametric data between groups was analyzed by Pearson Chi-Square analysis and Ficher's Exact Test (it was used because observed values were below the expected values). Parametric data were presented as minimum, maximum and mean±standard deviation and statistical significance of parametric data between the groups was analyzed by independent student t-test. The result was considered significant if two-tailed *P* value is below 0.05 (*P*<0.05) (Table 1).

**Table 1:** Data according to groups.

	Group 1 ( n=161) (On-pump CABG)	Group 2 ( n=95) (Off-pump CABG)	*P* value
Age (±SD) (year)	62.8 ±9.6	62.2± 9.8	0.620^T^
Gender (Male)	105 (% 65.2)	59 (% 62.1)	0.693^P^
Smoking	70 (% 43.5)	37 (% 38.9)	0.478^P^
COPD	36 (% 22.4)	32 (% 33.7)	*0.048^P^
Hypertension	134 (% 83.2)	74 (% 77.9)	0.291^P^
PAD	9 ( % 5.6)	3 ( % 3.2)	0.544^F^
Preoperative thrombocyte count	259.6±90.9	253.8±69.1	0.589^T^
Preoperative leukocyte count	8.14±5.24	8.39±2.15	0.659^T^
Preoperative stroke history	11 (% 6.8)	7 (% 7.4)	0.871^P^
Diabet oral a/d	48 (% 29.8)	30 (% 31.6)	0.927^P^
Parenteral a/d	28 (% 17.4)	15 (% 15.8)	
Weight (kg)	78.5±13.4	77.6±12.9	0.578^T^
BMI	29.6±5.1	29.8±5	0.784^T^
Ejection Fraction	53.8±9.7	54.6±8.7	0.514^T^

T =P value as Student-t test result;

P =P value as Pearson Qi-square test result;

F =Fischer’s Exact Test was used because observed values were below the expected values; CABG=coronary artery bypass grafting; COPD=chronic obstructive pulmonary disease; CAD=coronary artery disease; BMI=body mass index

## RESULTS

### Subjects characteristics

Distribution of age of all study participants was minimum (min) 29 years (y) and maximum (max) 89 years (mean±standard deviation 62.6±9.6 y). Of these subjects, 164 (64.1%) were male and 92 (35.9%) were female. It was observed that BMI was min 18.3 and max 50.2 (mean±standard deviation 29.6±5) kg/m^2^. The number of patients with hypertension (HT) was 208 (81.3%) and the number of patients receiving antidiabetic agent was 121 (47.3%). There were 107 (41.8%) smokers and 68 (26.6%) patients with COPD. The number of patients with history of stroke before surgery was 18 (7%), with right pleural effusion was 5 (2%), left pleural effusion was 21 (8.2%), and with bilateral pleural effusion was 20 (7.8%). Among study participants, the number of patients who underwent CABG together with CPB was determined to be 161 (62.8%) and the number of patients who underwent CABG via beating heart technique was determined to be 95 (37.2%). Some data about participants were shown at Table 1 and Table 2.

**Table 2:** Data according to groups as a postoperative.

	Group 1 ( n=161) (On-pump CABG)	Group 2 ( n=95) (Off-pump CABG)	*P* value
Numbers of grafts	3.6±0.8	2.6±0.9	<0.001^T^
Pleural Effusion			
Right	3 (% 1.9)	2 (% 2.1)	1^F^
Left	19 (% 11.8)	2 (% 2.1)	*0.008^F^
Bilateral	12 (% 7.5)	8 (% 8.4)	0.812^F^

T =P value as Student-t test result;

P=P value as Pearson Qi-square test result;

F =Fischer’s Exact Test was used because observed values were below the expected values; CABG=coronary artery bypass grafting; COPD=chronic obstructive pulmonary disease; CAD=coronary artery disease; BMI=body mass index

### Group characteristics

In the males of Group 1 it was determined that the number of patients with postoperative right pleural effusion was 3 (2.9%), with left pleural effusion was 11 (10.5%), and with bilateral pleural effusion was 8 (7.6%). The mean age (±standard deviation) was 62.2±10.3 y, the mean (±standard deviation) BMI was 28.3±4.6 kg/m^2^, the mean (±standard deviation) preoperative EF was 52.6±9.42, the mean (±standard deviation) bypass graft performed in CABG was 3.6±0.8, and the number of patients with history of CVA before surgery was 9 (8.6%). It was observed that there were 65 (61.9%) smokers, 81 (77.1%) hypertensive patients, 28 (26.7%) patients with COPD, 7 (6.7%) patients with PAD, 27 (25.7%) patients receiving oral antidiabetic agent and 12 (11.4%) patients receiving parenteral antidiabetic agent. The mean (±standard deviation) preoperative leukocyte count was 8.27±6.2 and the mean (±standard deviation) preoperative thrombocyte count was 248.6±75.4.

**Fig. 1:**
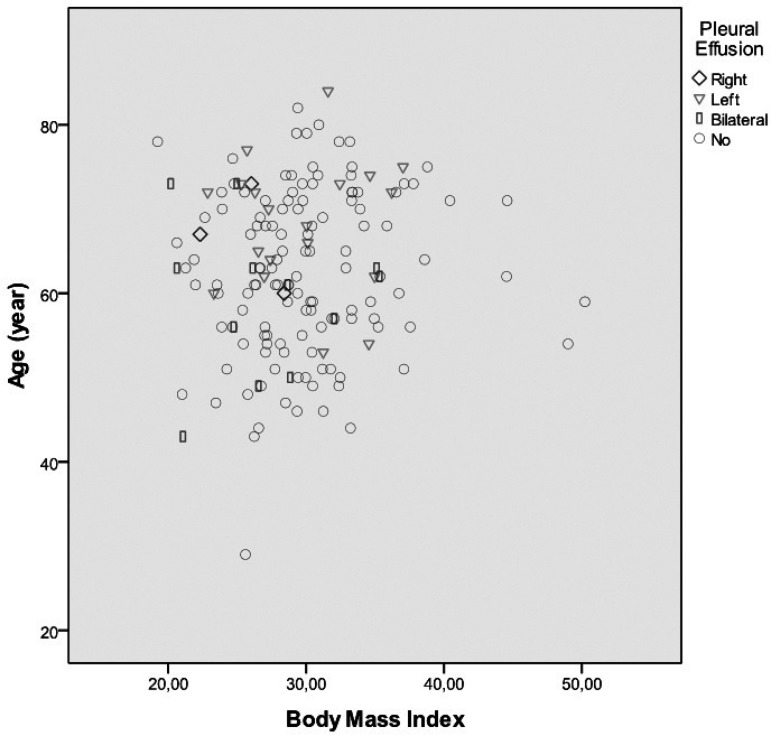
Pleural effusion dissociation graphic according to age and body mass index in group 1.

In the females of Group 1, it was determined that the number of patients with postoperative right pleural effusion was 0 (0%), with left pleural effusion was 8 (14.3%), and with bilateral pleural effusion was 4 (7.1%). The mean age (±standard deviation) was 64.05±8 y, the mean (±standard deviation) BMI was 31.9±5.1 kg/m^2^, the mean (±standard deviation) preoperative EF was 56.1±9.9, the mean (±standard deviation) bypass graft performed in CABG was 3.6±0.8, and the number of patients with history of CVA before surgery was determined to be 2 (3.6%). It was observed that there were 5 (8.9%) smokers, 53 (94.6%) hypertensive patients, 8 (14.3%) patients with COPD, 2 (3.6%) patients with PAD, 21 (37.5%) patients receiving oral antidiabetic agent, and 16 (28.6%) patients receiving parenteral antidiabetic agent. The mean (±standard deviation) preoperative leukocyte count was 7.9±2.5 and the mean (±standard deviation) preoperative thrombocyte count was determined to be 280.3±112.5 (Figure 1).

In the males of Group 2, it was determined that the number of patients with postoperative right pleural effusion was 1 (1.7%), with left pleural effusion was 1 (1.7%), and with bilateral pleural effusion was 5 (8.5%). The mean (±standard deviation) age was 61.5±9.5 y, the mean (±standard deviation) BMI was 28.1±3.8 kg/m^2^, the mean (±standard deviation) preoperative EF was 56.3±7.5, the mean (±standard deviation) bypass graft performed in CABG was 2.7±1 and the number of patients with history of CVA before surgery was 5 (8.5%). It was observed that there were 33 (55.9%) smokers, 42 (71.2%) hypertensive patients, 18 (% 30.5) patients with COPD, 2 (3.4%) patients with PAD, 19 (32.2%) patients receiving oral antidiabetic agent and 3 (5.1%) patients receiving parenteral antidiabetic agent. The mean (±standard deviation) preoperative leukocyte count was 8.5±2.1 and the mean (±standard deviation) preoperative thrombocyte count was 256.7±71.8.

In the females of Group 2, it was determined that the number of patients with postoperative right pleural effusion was 1 (2.8%), with left pleural effusion was 11 (2.8%) and with bilateral pleural effusion was 3 (8.3%). The mean (±standard deviation) age was 63.3±10.2 y, the mean (±standard deviation) BMI was 32.4±5.5 kg/m^2^, the mean (±standard deviation) preoperative EF was 51.8±10, the mean (±standard deviation) bypass graft performed in CABG was 2.5±0.9, and the number of patients with history of CVA before surgery was determined to be 2 (5.6%). It was observed that there were 4 (11.1%) smokers, 32 (88.9%) hypertensive patients, 14 (38.9%) patients with COPD, 1 (2.8%) patient with PAD, 11 (30.6%) patients receiving oral antidiabetic agent, and 12 (33.3%) patients receiving parenteral antidiabetic agent. The mean (±standard deviation) preoperative leukocyte count was 8.2±2 and the mean (±standard deviation) preoperative thrombocyte count was 249±65.

**Fig. 2:**
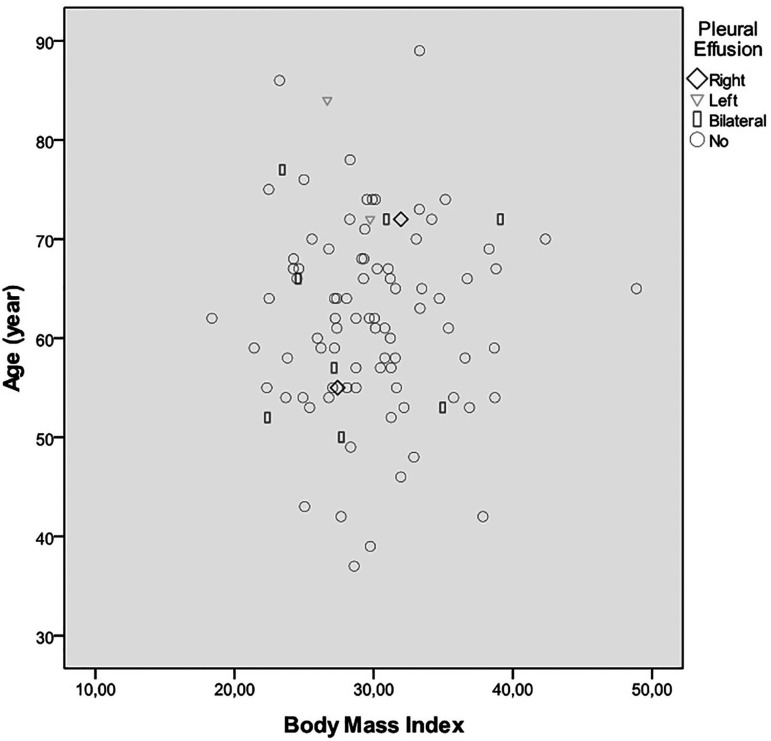
Pleural effusion dissociation graphic according to age and body mass index in group 2.

The patients were followed until hospital discharge for pleural effusion (very early period). Right pleural effusion was determined in six, left pleural effusion was determined in 21 and bilateral pleural effusion was determined in 20 patients in very early period (Figure 2).

## DISCUSSION

Conventional CABG is performed by using cardiopulmonary bypass (CPB) device and called as on-pump CABG, whereas CABG performed without CPB is called as off-pump CABG. On-pump CABG is agreed as the gold standard, but this method has some physiological effects. These effects include thrombocytopenia, activation of complement system, immune suppression, and inflammatory response that leads to organ dysfunction. There are two basic causes of perioperative pleural effusion following CABG. The first is pleural effusion due to atelectasis that results from diaphragm dysfunction. It is known that local cold application (slash) during CABG is directly associated with diaphragm paralysis and atelectasis. Correlation has been demonstrated between size of atelectasis and amount of effusion^[4]^. The second is the pleural effusion due to bleeding that occurs after harvesting internal mamarian artery. Pleural effusion occurs during and after bleeding, which results from trauma to parietal pleura during IMA harvesting. Slash was applied on all participants who underwent on-pump CABG surgery in our study. We tried to escape from adverse effects of slash by using only top of the heart and hood which it is save from phrenic injury. Our study did not include the patients who present diaphragm paralysis.

Pleural effusion may also develop due to congestive heart failure that occurs after CABG^[11]^. Decrease in cardiac output after surgery may lead to pulmonary edema and bilateral pleural effusion. All of participants in our study have no congestive heart failure. Pleural infection in early period, pulmonary embolus and surgery-related chylothorax as well can be considered among causes of pleural effusion^[5]^. There are studies demonstrating that some intraoperative techniques reduce pleural effusion. Gullu et al.^[12]^ reported that preserving pleural integrity while harvesting internal mammary artery reduces postoperative pain and the incidence of atelectasis and pleural effusion. It has been emphasized that using LIMA in coronary bypass surgeries enhances effusion as compared to using saphenous vein alone. In our study, LIMA was harvested and left pleural space was opened in all patients.

Many researchers including Burgess et al. stated that harvesting ITA (internal thoracic artery) during CABG surgeries increases complications and makes additional contribution to postoperative pulmonary dysfunction^[13-15]^. Bonacchi et al.^[10]^ indicated chest tube insertion and pleural injury while preparing ITA graft as the reasons for postoperative poor pulmonary function, which seems to support the findings of some researchers^[10,13,16,17]^.

There are studies demonstrating that inserting thoracic drain through intercostal space negatively influence patient comfort in early postoperative period and, in addition, increases the incidence of atelectasis and pleural effusion that occur due to chest wall trauma^[16-19]^. Contributing to some studies, Wimmer-Greinecker et al. reported that restriction in patient's movements until the removal of chest tubes causes decrease in pulmonary functions due to restricted inspiratory capacity^[17-19]^. There are studies stating that inserting subxiphoidal drain for pleural drainage is as effective as intercostal drains. In accordance with our clinical protocol, subxiphoidal chest tube was inserted in all patients aiming at elimination of unfavorable effects of intercostal drains. We had placed the chest tubes at subxiphoidal localization in all surgical procedures.

There are publications demonstrating that the incidence of pleural effusion and atelectasis between the 2^nd^ and 5^th^ postoperative days is significantly higher in the patients whose pleura was opened during surgery as compared to the patients whose pleura was not opened^[20]^. Rolla et al.^[21]^ emphasized that there was no increase in the incidence of atelectasis or pleural effusion between the 2^nd^ and 5^th^postoperative day with opening the pleura in the patients who underwent LIMA harvesting. Lim et al.^[22]^, Atay et al.^[23]^, and Oz et al.^[16]^ stated that atelectasis and pleural effusion are significantly more prevalent in the patients, in whom pleura was opened intraoperatively.

Atelectasis may be prevalent after CABG due to paralysis/paresis of diaphragm. Fedullo et al. demonstrated left diaphragm dysfunction after CABG in 16% of the patients via US^[6]^. Application of local cold cardioplegia causes phrenic nerve paresis as well as left inferior lobe atelectasis and increase in effusion between the 2^nd^ and 28^th^ postoperative day^[7,8]^. In a 30-patient study, Vargas et al.^[4]^ demonstrated 87% atelectasis via computed tomography on the 2^nd^ day after CABG. In the same study, they emphasized that there is significant correlation between the degree of atelectasis and pleural effusion between the 2^nd^ and 7^th^ postoperative day. In our study, we had applied respiratory physiotherapy on all patients from extubation until hospital discharge.

There are studies demonstrating that the incidence of pleural effusion is 5-11% in the case of pleura is not opened but increases to 20-50% in the case of pleura is opened during IMA harvesting^[9,10]^. It has been stated that the incidence of extensive pleural effusion that needs intervention during early postoperative period (first 7 postoperative days) is 0.5-8.5%^[3,24,25]^. Heidecker et al.^[5]^ reported that the most common causes of pleural effusion in the postoperative early period (first 7 days) are IMA harvesting, diaphragm dysfunction and atelectasis.

## CONCLUSIONS

In the present study, we compared incidence of pleural effusion in the 7 postoperative days between on-pump CABG and the CABG performed using CPB. Left pleural effusion was encountered to be lower in Group 2 (off-pump). The difference was found to be statistically significant (*P*<0.05, *P*=0.006). The conspicuous point is; right, left and bilateral pleural effusion was less prevalent in Group 2 although the number of patients with COPD, which is a factor that enhances atelectasis hence pleural effusion, was higher in Group 2. Under the light of these results, it can be said that left pleural effusion is less prevalent in the patients who underwent off-pump CABG as compared to the patients who underwent on-pump CABG. We believe that these data should be verified with larger case series.

### Study limitations

In order to create a homogenous group; dialysis patients or the patients with creatinine level higher than 2gr/dl, patients with aortic pathology detected during surgery and thereby surgery procedure was changed, patients who underwent emergency surgery, patients that underwent redo-CABG, patients with postoperative lower respiratory tract infection, patients who developed postoperative diaphragm paralysis, patients who were re-explored because of drainage, and patients who died were not included in the study. Moreover, patients who participate in the study are Caucasians.

**Table:** 

**Authors’ roles & responsibilities**	
MO	Final approval of the manuscript; manuscript writing or crit-ical review of its contents
FA	Final approval of the manuscript; manuscript writing or crit-ical review of its contents
